# Sex-Specific Cognitive Deficits Following Space Radiation Exposure

**DOI:** 10.3389/fnbeh.2020.535885

**Published:** 2020-09-16

**Authors:** Vipan K. Parihar, Maria C. Angulo, Barrett D. Allen, Amber Syage, Manal T. Usmani, Estrella Passerat de la Chapelle, Amal Nayan Amin, Lidia Flores, Xiaomeng Lin, Erich Giedzinski, Charles L. Limoli

**Affiliations:** Department of Radiation Oncology, University of California, Irvine, Irvine, CA, United States

**Keywords:** space radiation, sex specific, cognitive deficit, microglia, HMGB1, TLR4, neuroinflammation

## Abstract

The radiation fields in space define tangible risks to the health of astronauts, and significant work in rodent models has clearly shown a variety of exposure paradigms to compromise central nervous system (CNS) functionality. Despite our current knowledge, sex differences regarding the risks of space radiation exposure on cognitive function remain poorly understood, which is potentially problematic given that 30% of astronauts are women. While work from us and others have demonstrated pronounced cognitive decrements in male mice exposed to charged particle irradiation, here we show that female mice exhibit significant resistance to adverse neurocognitive effects of space radiation. The present findings indicate that male mice exposed to low doses (≤30 cGy) of energetic (400 MeV/n) helium ions (^4^He) show significantly higher levels of neuroinflammation and more extensive cognitive deficits than females. Twelve weeks following ^4^He ion exposure, irradiated male mice demonstrated significant deficits in object and place recognition memory accompanied by activation of microglia, marked upregulation of hippocampal Toll-like receptor 4 (TLR4), and increased expression of the pro-inflammatory marker high mobility group box 1 protein (HMGB1). Additionally, we determined that exposure to ^4^He ions caused a significant decline in the number of dendritic branch points and total dendritic length along with the hippocampus neurons in female mice. Interestingly, only male mice showed a significant decline of dendritic spine density following irradiation. These data indicate that fundamental differences in inflammatory cascades between male and female mice may drive divergent CNS radiation responses that differentially impact the structural plasticity of neurons and neurocognitive outcomes following cosmic radiation exposure.

## Introduction

Galactic cosmic rays (GCR) and energetic solar particles pose a range of adverse health risks to astronauts. GCR consist of high-energy protons (85%), helium (14%), and other high-energy nuclei (HZE ions of *Z* ≤ 26), while solar energetic particles consist primarily of protons derived from solar flares and coronal mass ejections (Nelson, 2016). Measurements of the radiation fields in space have provided considerable information concerning the types, fluences, and energies of charged particles that contribute to the expected doses astronauts would incur within and beyond the Earth’s protective magnetosphere (Nelson, 2016). Based on the measured dose rates and mission duration for a roundtrip mission to Mars, total doses are not expected to exceed 0.4–5 Gy, where the majority (~80%) of whole-body exposures will be due to lighter particles (e.g., protons and helium ions; Nelson, 2016).

Importantly, acute and chronic tissue alterations arise from the damaging effects of highly energetic charged particles that penetrate the spacecraft and traverse the tissues of the body (Cucinotta, 2014; Cucinotta and Cacao, 2019). Central nervous system (CNS) complications arise due to these damaging particle traversals that leave complex cellular damage along tracks of high radical density (Cucinotta, 2014). Terrestrial-based simulations of the space radiation environment have provided invaluable information concerning the biological effects of cosmic rays and have begun to identify how such exposures can compromise the functionality of the CNS (Cucinotta, 2014). Several studies have shown that simulated GCR exposures compromise dendritic complexity (Allen et al., 2015), cause long-term suppression of glutamatergic transmission and NR2A receptor levels in the hippocampus (Parihar et al., 2015b), and elicit persistent alterations in long-term potentiation and synaptic plasticity (Parihar et al., 2018) and increases in autophagy and prolonged oxidative stress (Hinzman et al., 2018).

Many of the foregoing changes portend potential neurobehavioral decrements, and significant past work with rodents has demonstrated that whole-body exposure to charged particles can disrupt behavioral performance that can be linked to impairments in the hippocampus (Britten et al., 2001, 2016; Cacao et al., 2018; Carr et al., 2018), amygdala (Rabin et al., 2014; Parihar et al., 2016), basal forebrain (Britten et al., 2014), mPFC (Britten et al., 2014, 2016; Parihar et al., 2015a), and other regions (Britten et al., 2014; Davis et al., 2014, 2015; Parihar et al., 2015a, 2016). Individual animals often exhibit deficits in multiple behavioral paradigms (Parihar et al., 2015b, 2016, 2018; Britten et al., 2020), and many of these decrements transpiring over 6–15 weeks have been found to coincide with a marked structural plasticity of neurons and glia and elevated neuroinflammation (Parihar et al., 2015b, 2018). The latter effect is particularly prominent, and increased yields of activated microglia found as long as 1 year after exposure provide a chronic “footprint” of radiation injury (Parihar et al., 2018). Importantly, in a recent study in which male mice were exposed to low doses of helium ions (^4^He), chronic inflammation was associated with persistent (1 year) and significant decrements in cognition that were coincident with network level disruptions in neurotransmission (Raber et al., 2018).

Radiation-induced cognitive dysfunction is a multifaceted disorder caused by elevated oxidative stress, neuroinflammation, declines in neurogenesis, and a degradation of neuronal structure and synaptic integrity (Poulose et al., 2014; Tseng et al., 2014; Allen et al., 2015; Parihar et al., 2015b, 2018; Acharya et al., 2016; Seawright et al., 2017; Carr et al., 2018; Hinkle et al., 2019). While many mechanisms influence these dynamic processes, microglia play an active role in reshaping the connective landscape of the brain by selectively pruning dendritic architecture and synapses in the irradiated or otherwise compromised brain (Lumniczky et al., 2017; Krukowski et al., 2018b; Rosi, 2018; Hinkle et al., 2019; Liu et al., 2019). In support of this, two recent studies have determined that elimination of microglia facilitates the functional recovery of the irradiated brain. Following higher-dose x-irradiation (Acharya et al., 2016) or lower-dose ^4^He irradiation (Acharya et al., 2016; Krukowski et al., 2018a; Allen et al., 2020), data showed an improved cognitive function, reduced inflammation, and a preservation of certain morphologic parameters in microglia-depleted brains. Once activated, microglia initiate and participate in pro-inflammatory signaling pathways in the brain (Fellner et al., 2013; Fiebich et al., 2018; York et al., 2018). One such pathway includes high mobility group box protein 1 (HMGB1), a ubiquitous nuclear protein that can be released by microglia (and other immune cells) to bind and activate the transmembrane, pattern recognition receptors for advanced glycation end products (RAGE), and toll-like receptor 4 (TLR4) on neurons and glia (Bianchi et al., 2017; Paudel et al., 2018). HMGB1 and TLR4 have attracted recent attention due to their roles in mediating traumatic brain injury (Fujita et al., 2016; Webster et al., 2019), Alzheimer’s pathology (Fujita et al., 2016), neuroinflammatory conditions (Trotta et al., 2014), epileptogenesis (Paudel et al., 2018), and cognitive impairments (Paudel et al., 2018), thereby providing logical targets for intervention (Fujita et al., 2016; Okuma et al., 2019). These prior findings also suggested the potential importance of these inflammatory pathways in regulating the response of the irradiated brain, although purported sex-specific differences remained largely unexplored.

The foregoing gap in knowledge has resulted largely from the paucity of studies conducted in female mice. Studies in male mice have pointed to the vulnerability of the male rodent brain to exposures from ^4^He and other charged particle types (Rabin et al., 2014; Parihar et al., 2018; Raber et al., 2018). Certain work, however, has begun to shed light on differences between the sexes regarding neurobehavioral susceptibility to charged particle irradiation (Villasana et al., 2011; Allen et al., 2015; Krukowski et al., 2018b; Paudel et al., 2018; Kiffer et al., 2019). It is increasingly recognized that sex can significantly influence neural responses in development (Helmstaedter et al., 1999; Hanamsagar et al., 2017; Doran et al., 2019), aging (Christakou et al., 2009), disease (Mangold et al., 2017), and injury (Spychala et al., 2017). For example, phenotypic differences between male and female microglia may account for sex differences in neurological disease susceptibility and outcomes in several neurodegenerative disorders (Trotta et al., 2014; Villa et al., 2018). The impact of sex on radiation effects and in particular on female mice has been historically underreported (Narendran et al., 2019). Recently, several groups have clearly shown that radiation exposure affects males and females differently (Villasana et al., 2010, 2011; Krukowski et al., 2018b; Hinkle et al., 2019; Kiffer et al., 2019; Liu et al., 2019). Furthermore, increasing evidence suggests sex differences in microglial activation, synaptic modifications, and cognition following high-LET particle irradiation (Carr et al., 2018; Krukowski et al., 2018b; Kiffer et al., 2019; Liu et al., 2019).

These studies have determined that similar irradiation paradigms elicit increased neuroinflammation, dendritic spine loss, and more extensive cognitive deficits in male compared to female mice (Krukowski et al., 2018b; Hinkle et al., 2019; Liu et al., 2019). In contrast to these sex-dependent changes, another study found that hippocampus-dependent contextual fear conditioning was impaired in female compared to male mice following ^56^Fe irradiation (Villasana et al., 2010). Work from this same group also determined that overexpression of Apolipoprotein-E protected female mice from radiation-induced cognitive deficits, but not in males (Villasana et al., 2011). Here we sought to elucidate further potential sex-dependent differences in behavioral responses and the underlying mechanisms following cosmic radiation exposure. Our findings suggest that sex-dependent differences in the response to cosmic irradiation can be linked to an attenuation of neuroinflammation and oxidative stress in female mice, providing a plausible explanation for their resistance to the adverse neurocognitive effects of ^4^He exposure.

## Materials and Methods

### Animals and Irradiation

All animal procedures were carried out in accordance with National Institutes of Health and Institutional Animal Care guidelines and were approved by the Institutional Animal Care and Use Committee at the University of California, Irvine and NASA Space Radiation Laboratory (NSRL) at Brookhaven National Laboratory. Six-month-old male and female C57BL/6J mice acquired from the Jackson Laboratory (stock #000664) were used for the behavior [Novel Object Recognition (NOR), Object-in-Place (OiP), and Temporal Order (TO)] studies and immunohistochemical analysis. For the structural analysis of neurons expressing enhanced green fluorescent protein (EGFP), 6-month-old male and female transgenic mice [strain Tg(Thy1-EGFP) MJrsJ, Stock No. 007788, the Jackson Laboratory, CT, USA] harboring the Thy1-EGFP transgene were used. Charged particles (^4^He) at 400 MeV/n were generated and delivered at the NSRL at Brookhaven National Laboratory. All animals received whole-body irradiation at doses of either 5 or 30 cGy delivered at a dose rate of 5 cGy/min. At these low doses of cosmic radiation, significant changes in body weight were not found. The NSRL physics staff performed all radiation dosimetry and confirmed spatial beam uniformity. Additional details regarding the characterization and configuration of the NSRL beam line have been included in the Supplementary Materials and have recently been described in considerable detail (La Tessa et al., 2016).

### Behavioral Testing

Twelve weeks after irradiation, mice were subjected to NOR, OiP, and TO tasks to quantify hippocampal-, medial prefrontal cortex (mPFC)-, and/or perirhinal cortex (PRC)-dependent cognitive tasks. NOR is a measure of preference for novelty, which relies on intact mPFC function, while OiP is a test of associative recognition memory that depends on interactions between the hippocampus, mPFC, and PRC functions (Barker et al., 2007; Barker and Warburton, 2015). The TO task discriminates previously explored objects in terms of their relative recency; thus, TO memory can be defined as the ability of mice to recall a past experience encountered, which depends on interactions between the mPFC and PRC (Barker et al., 2007; Barker and Warburton, 2015). All behavior tests were performed 12–15 weeks post irradiation during the day with illumination at 915 lux as described previously (Barker et al., 2007; Barker and Warburton, 2011, 2015; Parihar et al., 2018). Data were collected and analyses were performed by independent observers who were blinded to the identity of animal groups.

$$\left(\frac{\text{Time spent exploring novel object}}{\text{total exploration time}}-\frac{\text{Time spent exploring familiar object}}{\text{total exploration time}}\right)\times 100$$

To quantify preference or indifference for exploring novelty, a discrimination index (DI) was calculated as below. A positive score indicates a preference, or more time exploring the novel object, while a negative score indicates indifference, or more time exploring the familiar object. All objects used in this study have equal innate preferences and can be discriminated easily by control mice (unirradiated mice). Objects were chosen to be matched for size, easily cleanable, and made of a non-porous material. Objects used in this study were consistent in height and volume but were different in shape and appearance. We have extensively screened the objects in our laboratory to meet these criteria. None of the objects produced a fear response and were explored adequately during the testing sessions (at least 7–8 s per object).

To elaborate, all the objects used in behavioral tests were made of material (glass, hard plastic) that a mouse cannot damage by chewing or scratching. To facilitate discrimination, objects were different albeit with relatively similar dimensions (7**–**9 cm, length; 4–5 cm, width), area (30–50 cm^2^), and degree of complexity (shape, size, texture, color, brightness, and pattern) in order to minimize any potential induced object preference that may bias the results. Objects were differentiated by size and shape, such as large (7–9 cm tall), small (6–8 cm tall), and smooth (having a regular, cylindrical shape); color (blue, pink, red, yellow, and white); and complex (having sharp angles, curves, or extensions). Additionally, the objects were randomized during behavioral testing to minimize the chance that any characteristics inherent to the objects might affect preference. All objects were secured with magnets to prevent animals from moving them during exploration. Time spent sitting on an object was not counted towards exploration time. Additional details regarding open arena testing can be found in the Supplementary Materials.

### Immunohistochemistry, Confocal Microscopy, and Quantification

For immunohistochemical analysis, 15 weeks following ^4^He irradiation, mice (*n* = 5/group) were euthanized and perfused with 4% paraformaldehyde (Acros Organics), and brain tissues were processed for coronal sectioning using a cryostat (Leica Microsystems). Six serial sections (30 μm, every 10th section) were stained and used for the identification and quantification of activated microglia (CD68+ cells), HMGB1, and protein for TLR4 in the hippocampus. Analysis was performed using the surface tool [Imaris software suite (v7.6, Bitplane, Inc.)] using 3-D confocal microscopy (Parihar and Limoli, 2013; Parihar et al., 2015c; Acharya et al., 2016, 2017). Detailed methods and procedures regarding immunohistochemistry procedure and confocal microscopy are provided in Supplementary Materials.

### Morphometric Assessments of Neurons

For structural analyses of neurons, Thy1-EGFP transgenic mice were bred and genotyped to confirm the presence of the transgene. At 6 weeks post-irradiation, Thy1-EGFP-positive mice were perfused with 0.1 M PBS (pH 7.4) followed by 4% (wt/vol) paraformaldehyde. Brains were then dissected out, post-fixed in 4% (wt/vol) paraformaldehyde for 24 h, washed, and stored in PBS at 4°C until sectioning. Brains were serially sectioned at 100 μm on a cryostat (Leica), then cryoprotected at −20°C. The signal intensity of eGFP fluorescence is similar between hemizygous and homozygous Thy1-GFP mice and sufficient in both cases for rigorous morphometric determinations. We have previously shown that changes in dendritic complexity parameters following irradiation result from the impact of radiation on neuronal structure. Homozygous or hemizygous Thy1-EGFP mice express EGFP within neurons of the hippocampus and other brain regions, thereby providing a brightly fluorescent signal that greatly facilitates the micromorphometric analyses of neurons. The exceptional clarity of the fluorescent neurons in these mice provides an accurate, precise, and rigorous analysis and quantification of the complete dendritic tree. For dendritic analysis, paraformaldehyde-fixed 100-μm-thick hippocampus sections were prepared for the imaging neurons within the molecular (ML) and granular cell layers (GCL) of the dentate gyrus (reference to the Bregma, −2.0 to −2.8 mm) using confocal microscopy. For each cohort (*n* = 3), three sections per animal were scanned to generate *z-stacks* using a Nikon Eclipse Ti C2 microscope. Images comprising each *z-stack* (1,024 × 1,024 pixels) were acquired at (60×) over the entire dendrite tree at 0.1-μm increments (Parihar and Limoli, 2013; Parihar et al., 2015c; Montay-Gruel et al., 2019). Details regarding the reconstruction of neurons and the morphologic classification of spines have been described in the Supplementary Materials. A recent study has demonstrated that microglia play a role in radiation-mediated synaptic loss and identified CR3-dependent signaling as an underlying mechanism for the vulnerability of spine populations in male mice (Hinkle et al., 2019). The reduced dendritic complex and spine loss were limited to male mice, and spine density was unaltered by radiation in female mice.

### Statistical Analysis

**Figure 1:**
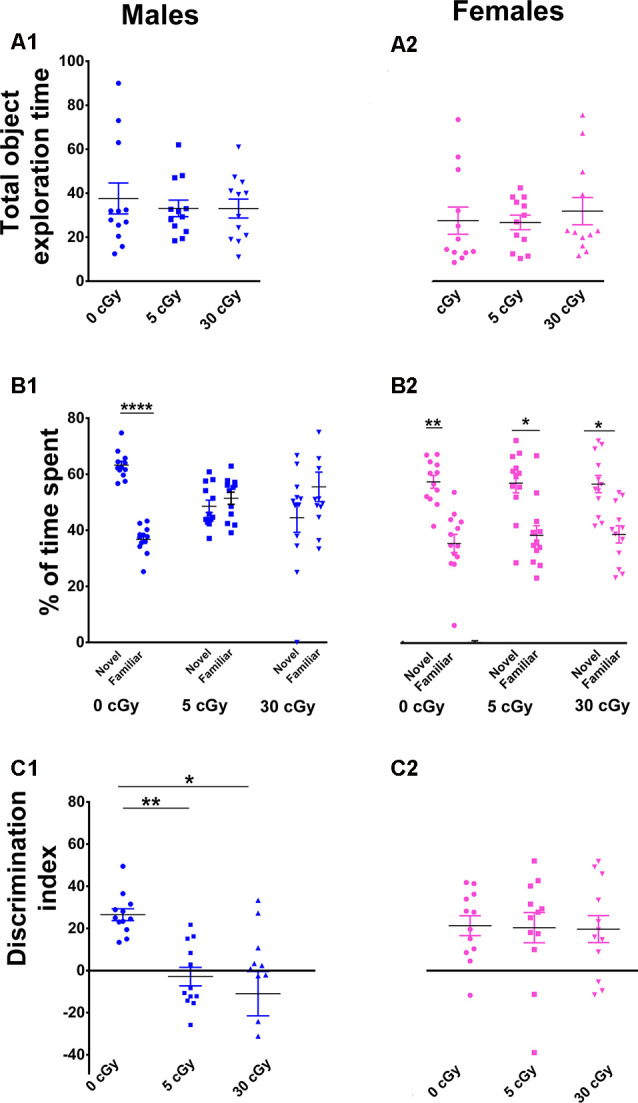
Exposure to ^4^He ions impairs Novel Object Recognition (NOR) in male but not female mice. Irradiation had no effect on total exploration time during the NOR task **(A1,A2)**. Irradiated male mice showed diminished preference for novelty **(B1)**, while irradiated female mice showed a higher preference for novelty **(B2)**. Two-way ANOVA on discrimination index (DI) revealed a significant interaction between sex and radiation (*p* = 0.048), as well as a significant sex effect (*p* < 0.0001), but no main effect of radiation (*p* = 0.10). *Post hoc* analysis showed that DI was reduced significantly in male mice following irradiation, but not in female mice **(C1,C2)**. Unlike males, female mice showed no indication of cognitive deficits following irradiation. *N* = 12 for each experimental group. Individual animals represented as dots; lines depict group means and SEM. *p < 0.05, **p < 0.01, ****p < 0.0001.

**Figure 2:**
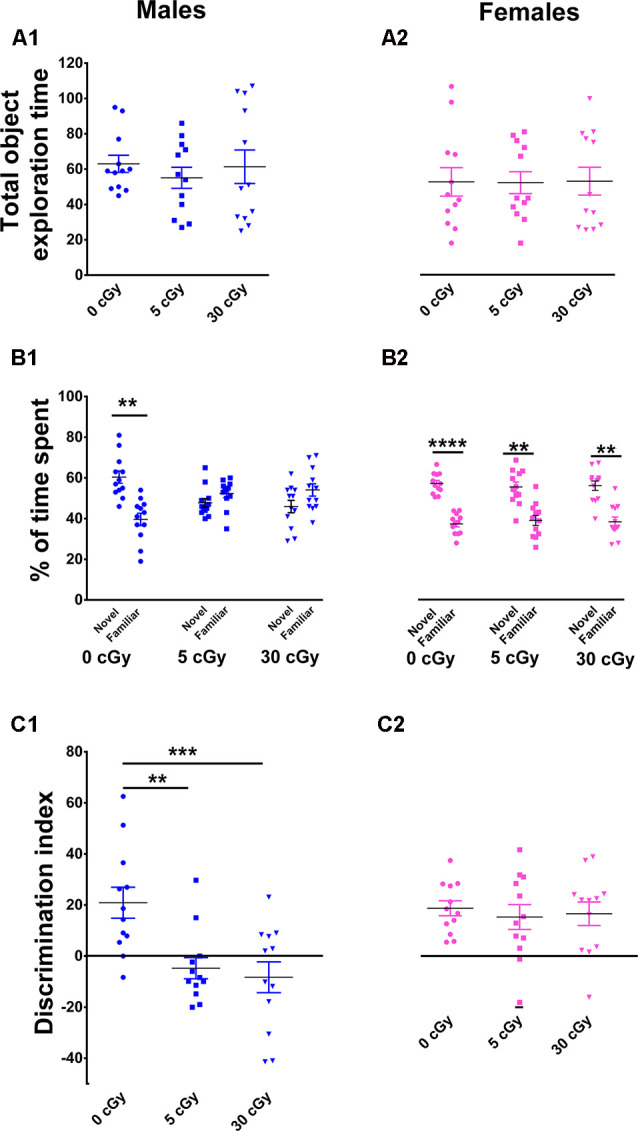
Object in place memory is impaired in male but not female mice after ^4^He irradiation. During the Object-in-Place (OiP) task, total exploration times were comparable between controls and irradiated cohorts **(A1,A2)**. Preference for novelty was reduced significantly following irradiation in male mice **(B1)**, but not in female mice **(B2)**. Two-way ANOVA revealed a significant sex × radiation interaction (*p* = 0.03), a significant radiation effect (*p* = 0.0001), and a significant sex effect (*p* = 0.04). *Post hoc* analysis showed that DI was significant reduced in male mice following irradiation, but not in female mice **(C1,C2)**. As opposed to males, irradiated female mice showed no signs of cognitive impairment. Individual animals represented as dots; lines depict group means and SEM. *N* = 12 for each experimental group. **p < 0.01, ***p < 0.001, ****p < 0.0001.

**Figure 3:**
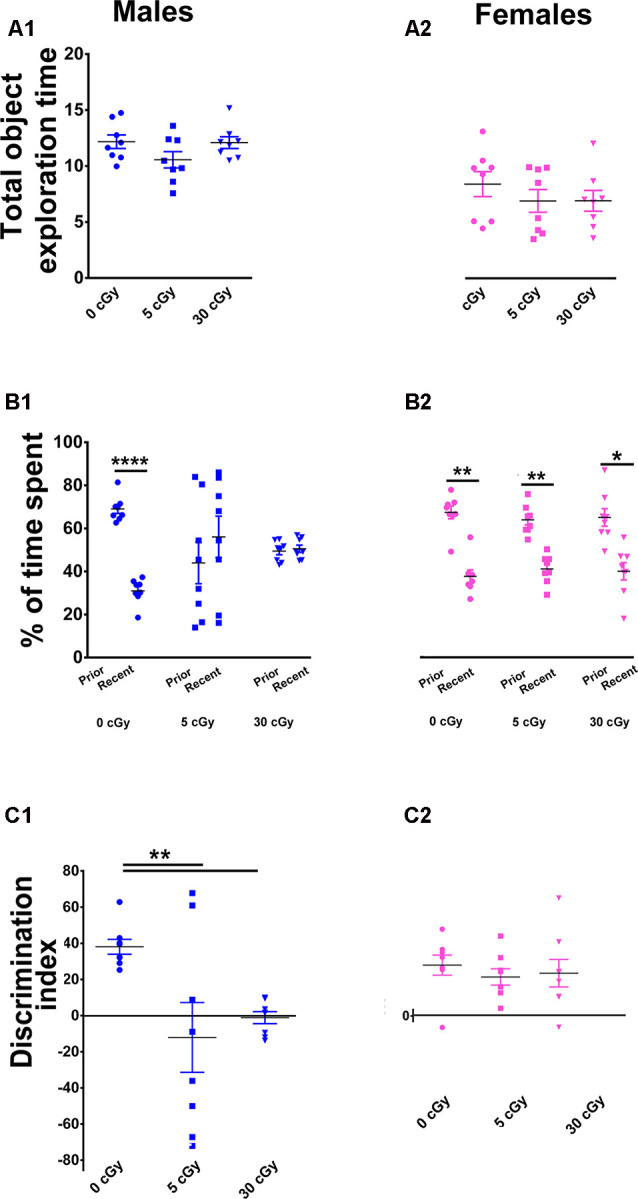
Irradiation with ^4^He ions causes deficits in recency memory in male but not female mice. Irradiation had no effect on total exploration time during the Temporal Order (TO) task **(A1,A2)**. In male mice, preference for novelty was reduced significantly following irradiation **(B1)**, while in female mice, no change was found **(B2)**. Two-way ANOVA revealed a significant sex × radiation interaction (*p* = 0.05), a significant radiation effect (*p* = 0.008), and a significant sex effect (*p* = 0.03). *Post hoc* analysis showed that DI was reduced significantly in male mice following irradiation, but not in female mice **(C1,C2)**. In contrast to males, irradiated female mice showed intact recency memory. Individual animals represented as dots; lines depict group means and SEM. *N* = 8 for each experimental group. *p < 0.05, **p < 0.01, ****p < 0.0001.

All data were analyzed, and figures created with GraphPad Prism v6.0 (GraphPad Software; La Jolla, CA, USA). All the behavioral, immunohistochemistry, and morphometric data were analyzed using two-way ANOVA considering sex and radiation as independent variables. When significant interaction effects were found, Bonferroni *post hoc* analyses were performed to elucidate the differences between groups within conditions. Given overall radiation effects in the absence of sex × radiation interactions, unpaired two-tailed Student’s *t*-tests were performed for female and male cohorts separately (Figures 5–8). Furthermore, a two-tailed paired Student’s *t*-test was used to evaluate the preferences for novelty (novel object or place location; Figures 1–3B1,B2). Data are depicted as the mean ± standard error of the mean (SEM) with individual data points overlaid; *p < 0.05, **p < 0.01, ***p < 0.001, ****p < 0.0001. Statistical significance was assigned at *p* ≤ 0.05.

**Figure 4:**
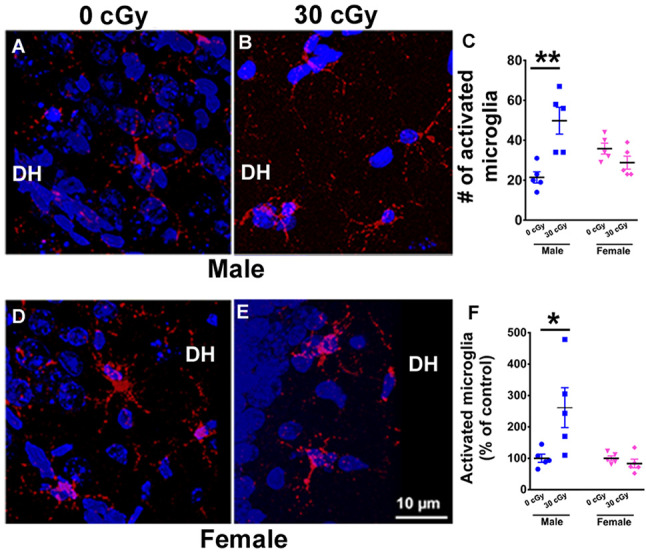
Microglial activation in mice 15 weeks following ^4^He ion irradiation. Representative confocal images showing activated microglia (CD68+, red) in the counterstained (blue) Dentate Hilus (DH) of the hippocampus for male **(A,B)** and female **(D,E)** mice. Two-way ANOVA demonstrated a significant interaction between irradiation and sex (*p* = 0.001), a significant radiation effect (*p* = 0.02), but no sex effect (*p* = 0.44). *Post hoc* analysis revealed a 2.5-fold increase in the number of activated microglia when compared to same-sex controls (*p* = 0.001, **C**). Female mice showed no change in the number activated microglia (*p* = 0.99, **C**). A similar two-way ANOVA on the percentage of activated microglia showed a significant interaction between irradiation and sex (*p* = 0.02), a significant radiation effect (*p* = 0.04), and a significant sex effect (*p* = 0.02). *Post hoc* analysis showed a significant increase in the percentage of activated microglia in the hippocampus of irradiated male mice when compared to same-sex controls (*p* = 0.02, **F**). Female mice showed no such change (*p* = 0.99, **F**). Percentages shown reflect values normalized to the average of all unirradiated controls, arbitrarily set to 100%. Data are expressed as the mean ± SEM, *N* = 5 for each experimental group. *p < 0.05, **p < 0.01 vs. unirradiated males.

**Figure 5:**
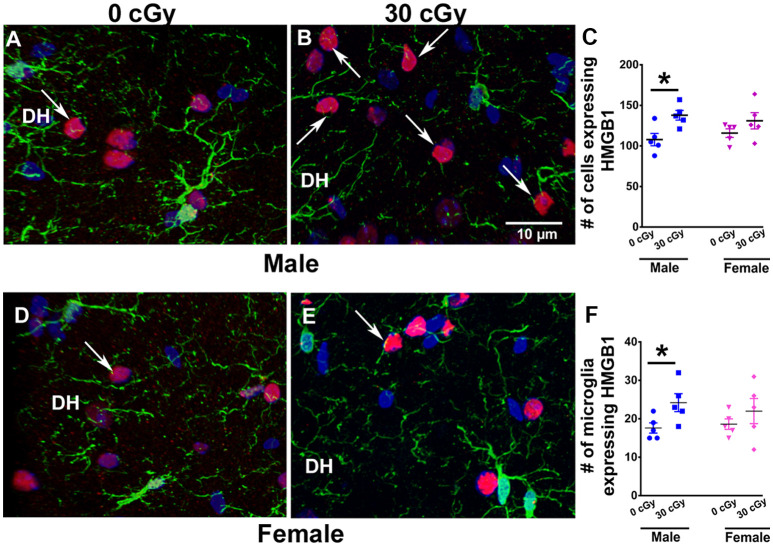
Enhanced expression of HMGB1 in male mice 15 weeks following ^4^He ion exposure. An example of HMGB1 expression in DH of the hippocampus is shown for male **(A,B)** and female **(D,E)** mice. Two-way ANOVA on total number of HMGB1 revealed no significant interaction between sex and irradiation (*p* = 0.33) and no significant effect of sex (*p* = 0.93) but did show a significant radiation effect (*p* = 0.008). Two-tailed unpaired student’s *t*-test showed a significant increase in the expression of HMGB1 in irradiated male mice (*p* = 0.01, **C**), while in female mice, the expression was unchanged (*p* = 0.21, **C**). Similarly, a significant increase in the number of microglia expressing HMGB1 in male mice was found (*p* = 0.04, **F**), while no such change was evident in the female mice (*p* = 0.36, **F**). HMGB1 (red), IBA1 (green), and nuclear staining (blue). Data are expressed as the mean ± SEM, *N* = 5 for each experimental group, *p < 0.05 vs. unirradiated males.

**Figure 6:**
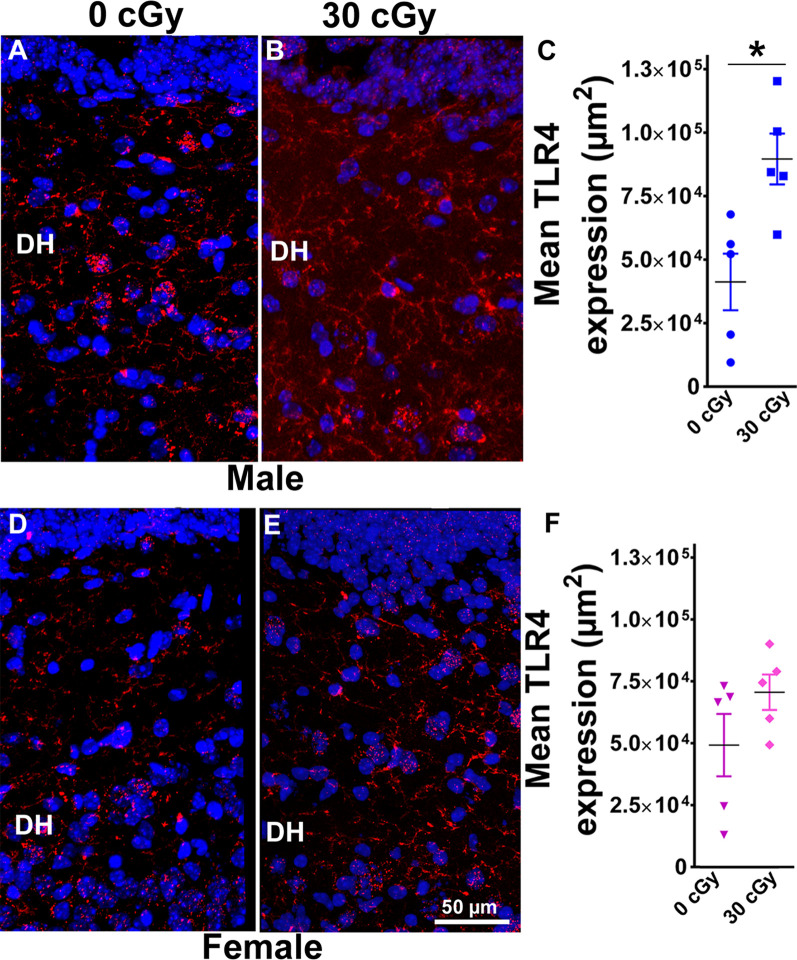
Enhanced expression of toll-like receptor 4 (TLR4) in male mice 15 weeks following ^4^He ion irradiation. Confocal images showing the expression of TLR4 in DH of the hippocampus is shown for male **(A,B)** and female **(D,E)** mice. Two-way ANOVA revealed no significant interaction between sex and irradiation (*p* = 0.33) and no significant effect of sex (*p* = 0.49) but did show a significant radiation effect (*p* = 0.01). Two-tailed unpaired Student’s *t*-test showed that irradiation significantly enhanced expression of TLR4 in male mice (*p* = 0.01, **C**), while the expression of TLR4 in female mice was unchanged (*p* = 0.17, **F**). Data are expressed as the mean ± SEM, *N* = 5 for each experimental group, *p < 0.05 vs. unirradiated males. TLR4 (red), nuclear staining (blue).

**Figure 7:**
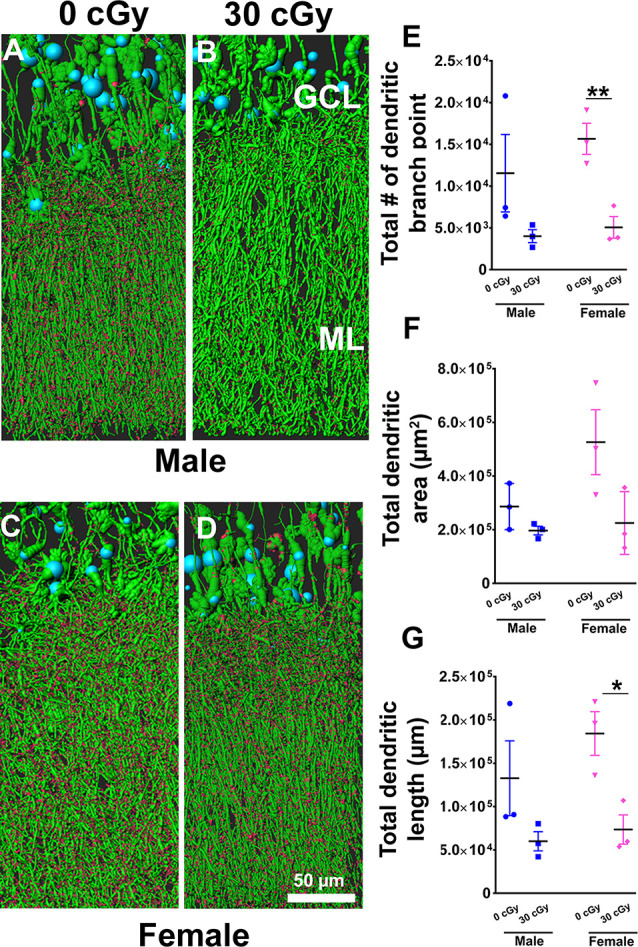
Dendritic complexity of dentate gyrus granular cell layer (GCL) neurons was altered in females not in males by ^4^He ion irradiation **(A–D)**. Examples of deconvoluted EGFP+ GCL neurons showing dendrites orientated vertically and traversing the molecular layer (ML) along with dendritic spines projecting into the ML. Two-way ANOVA (sex × radiation) performed on dendritic branch points **(E)**, area **(F)**, and length **(G)** confirmed no sex × radiation interaction (dendritic branch points, *p* = 0.57; area, *p* = 0.19; length, *p* = 0.50) and no main effect of sex (dendritic branch points, *p* = 0.35; area, *p* = 0.11, length, *p* = 0.26) but did show a significant effect of irradiation (dendritic branch points, *p* = 0.008; area; *p* = 0.03; length, *p* = 0.01). Radiation was found to decrease branch point and total dendritic length in female mice, but not male mice [branch point, *p* = 0.009 **(E)**; dendritic length, *p* = 0.02 **(G)**; unpaired *t*-tests]. Data are expressed as the mean ± SEM, *p < 0.05, **p < 0.01 unirradiated controls. *N* = 3 for each experimental group. Blue (cell body), green (dendrites), and red (dendritic spines).

**Figure 8:**
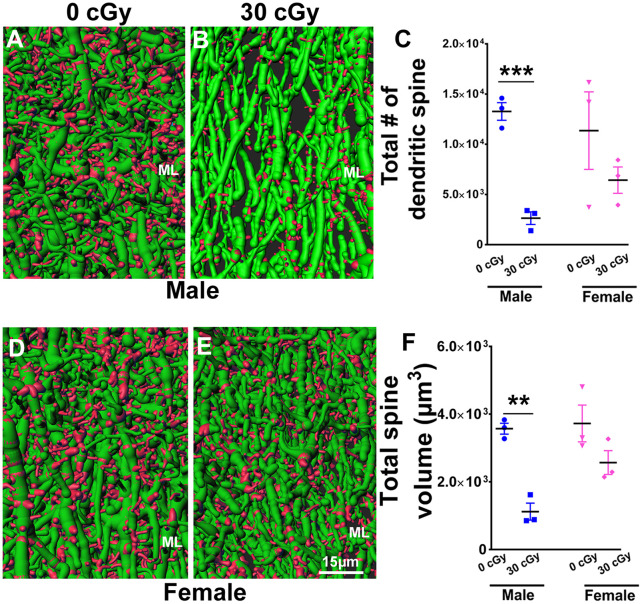
Dendritic spine numbers and volume are reduced following ^4^He ion irradiation in male mice. Representative images of 3D reconstructed dendritic segments (green) containing spines (red) are shown for male **(A,B)** and female **(D,E)** mice. Two-way ANOVA (sex × radiation) performed on dendritic spine number and spine volume confirmed no interaction between sex × radiation (spine number, *p* = 0.21; spine volume, *p* = 0.11) and no main effect of sex (spine number, *p* = 0.20; spine volume, *p* = 0.05) but did show a significant effect of irradiation (spine number, *p* = 0.006; spine volume, *p* = 0.001). Two-tailed unpaired Student’s *t*-test showed a significant effect of radiation on spine number and spine volume between irradiated male mice and controls (spine number, *p* = 0.0006, **C**; spine volume, *p* = 0.001, **F**). In female mice, dendritic spine number and volume were comparable between irradiated and control groups (spine number, *p* = 0.29, **C**; spine volume, *p* = 0.14, **F**). Data are expressed as the mean ± SEM, *N* = 3 for each experimental group. **p < 0.01, ***p < 0.001 vs. unirradiated controls.

## Results

### Sex-Specific Cognitive Decrements Following ^4^He Ion Irradiation

Past work has shown that male mice exposed to energetic charged particles of varying mass exhibited significant impairments on behavioral tasks known to interrogate the integrity of hippocampal and cortical circuitry (Parihar et al., 2015a,b, 2016, 2018). Present findings now indicate that females show a marked resistance to similar doses and do not exhibit the same level of behavioral deficits as observed in male mice following exposure to ^4^He ions. To assess the functional consequences of ^4^He ion particle exposure on the brain, 6-month-old male and female mice were tested 12–15 weeks post exposure using three distinct behavioral tasks that interrogate hippocampal, medial prefrontal, and perirhinal cortical function. Impairment in these brain regions manifests as an inability to discriminate a spatial, object, and/or temporal novelty. To quantify preference or indifference for exploring such novelty, a DI was calculated. A positive score indicates a preference, or more time exploring the novel object, while a negative score indicates indifference, or more time exploring the familiar object.

For the NOR test, a two-way ANOVA analysis on total time spent exploring both the objects revealed no significant interaction between sex and irradiation (*F*_(2,66)_ = 0.40, *p* = 0.67) as well no significant effect of sex (*F*_(1,66)_ = 2.67, *p* = 0.10) and irradiation (*F*_(2,66)_ = 0.15, *p* = 0.10). This confirms that exposure to ^4^He ion irradiation did not impair locomotor activity of the mice (Figures 1A1,A2). Furthermore, *t*-test revealed that both unirradiated male and female mice displayed a clear preference for novelty, evident by the greater percentage of time spent exploring the novel object (male 63%, *p* = 0.0001, Figure 1B1; female 60%, *p* = 0.001, Figure 1B2). However, while male mice irradiated with 5 or 30 cGy showed no preference for novelty (5cGy, 48%, *p* = 0.52; 30cGy, *p* = 0.31, 45%; Figure 1B1), female mice did not exhibit such impairments and retained the capability to distinguish the novel object (5cGy, 60%, *p* = 0.02; 30cGy, 60%, *p* = 0.01; Figure 1B2).

For the DI, a two-way ANOVA analysis revealed a significant sex × radiation interaction effect (*F*_(2,66)_ = 3.17, *p* = 0.048) and a sex effect (*F*_(1,66)_ = 23.61, *p* < 0.0001) but no radiation effect (*F*_(2,66)_ = 2.34, *p* = 0.10). *Post hoc* analysis showed that control male mice showed a high DI compared to their irradiated counterparts and showed a statistical group difference, demonstrated by a marked reduction in the preference to explore novelty (5cGy, *p* = 0.01; 30 cGy, *p* = 0.04; Figure 1C1). In contrast, irradiated female mice showed intact object recognition memory and displayed comparable DI to the control group (5cGy, *p* = 0.99, 30 cGy, *p* = 0.99; Figure 1C2). The preference for novelty, as indicated by the DI between sham irradiated male and female mice, was comparable (novelty preference, *p* = 0.99; DI, *p* = 0.99; see the Supplementary Materials and Supplementary Table S1). Hence, the observed differences between the sexes following irradiation were not confounded by sham-irradiated cohorts and were due to radiation-induced changes in male mice.

Following the NOR task, mice were habituated and tested on the OIP task, also known to be reliant on intact hippocampal, prefrontal, and perirhinal cortical function. In this case, functionally intact mice exhibit a preference towards objects that have been moved to a novel location. Again, a two-way ANOVA performed on total time explored revealed no main effect of sex (*F*_(1,66)_ = 1.22, *p* = 0.27) or radiation (*F*_(2,66)_ = 0.19, *p* = 0.82) as well as no significant interaction between sex and radiation (*F*_(2,66)_ = 1.22, *p* = 0.87). This indicates, as before, that exposure to ^4^He ions did not impair locomotor activity of the mice (Figures 2A1,A2). Furthermore, paired *t*-test revealed that controls in both cohorts displayed a clear preference for the objects moved to a novel location (male, 60%, *p* = 0.005, Figure 2B1; female, 61%, *p* = 0.0001, Figure 2B2) compared to objects that remained at the same (familiar) location during the test phase. The preference for novelty was abolished in irradiated male mice, evident by an equal tendency to explore objects at either location (5cGy, *p* = 0.28, 48%; 30cGy, *p* = 0.20, 46%; Figure 2B1). In contrast, the irradiated female cohorts spent the majority of time with the objects at novel locations (5 cGy, *p* = 0.006, 59%; 30 cGy, *p* = 0.002, 59%; Figure 2B2).

For the DI, a two-way ANOVA revealed a significant sex effect (*F*_(1,66)_ = 16.35, *p* = 0.0001), radiation effect (*F*_(2,66)_ = 6.15, *p* = 0.004), and significant sex × radiation interaction effect (*F*_(2,66)_ = 2.97, *p* = 0.03). *Post hoc* analysis revealed that control male mice had a high DI compared to their irradiated counterparts, where a statistical group difference was indicated by a marked reduction in the preference to explore novelty (5cGy, *p* = 0.01; 30 cGy, *p* = 0.001; Figure 2C1). In contrast, DI was comparable between controls and irradiated female mice (5cGy, *p* > 0.05; 30 cGy, *p* > 0.05; Figure 2C2). These results indicate that as opposed to irradiated male mice, exposure to ^4^He ions did not affect object in place memory in females 13 weeks post-exposure. The preference for novelty as shown by the DI was again comparable between the unirradiated male and female groups (novelty preference, *p* = 0.99; DI, *p* = 0.99; see the Supplementary Materials and Supplementary Table S1). These results indicate that differences in object in place memory following irradiation in male mice are not attributable to changes in sham irradiated mice.

Last, cohorts were subjected to a TO task where animals were familiarized with two sets of objects, 4 h apart. In this instance, mice with intact hippocampal, mPFC, and PRC function exhibit a preference for exploring the prior, rather than the more recent object (Barker et al., 2007; Barker and Warburton, 2011). Similar to the OiP task, two-way ANOVA found that the total time exploring both objects revealed no main effect of sex (*F*_(1,42)_ = 1.35, *p* = 0.25) or radiation (*F*_(2,42)_ = 0.15, *p* = 0.25) as well as no significant interaction between sex and radiation (*F*_(2,42)_ = 0.47, *p* = 0.63), indicating again that locomotor activity was not impaired by the ^4^He exposures (Figures 3A1,A2). Furthermore, paired *t*-test revealed that during the test phase of the TO task, the unirradiated mice in both cohorts displayed a clear preference for exploring the object presented earlier rather than more recently in time (male, 69%, *p* < 0.0001, Figure 3B1; female, 64%, *p* < 0.001, Figure 3B2). As with prior tests, the irradiated male mice showed a reduced capability to discriminate novelty (in this instance, “recency”) as evident by the reduced preference for objects presented earlier in time (5 cGy, *p* = 0.55, 44%; 30 cGy, *p* = 0.73, 49%; Figure 3B1). In contrast, the irradiated female mice showed a preference for exploring the object presented earlier in time, indicating no ^4^He-induced temporal order memory deficits (5cGy, *p* < 0.002, 61%; 30cGy, *p* = 0.02, 62%; Figure 3B2).

As found in earlier testing, for DI, two-way ANOVA revealed a significant sex effect (*F*_(1,42)_ = 5.17, *p* = 0.03), radiation effect (*F*_(2,42)_ = 5.35, *p* = 0.008), and significant sex × radiation interaction effect (*F*_(2,42)_ = 3.25, *p* = 0.05). *Post hoc* analysis showed that control male mice had a high DI compared to their irradiated counterparts, which showed a statistical group difference as demonstrated by a marked reduction in the preference to explore novelty (5cGy, *p* < 0.01; 30 cGy, *p* < 0.01; Figure 3C1). In the female groups, DI was comparable between control and irradiated mice, indicating again that ^4^He ion irradiation did not impair recency discrimination at 14 weeks post exposure (5cGy, *p* > 0.05; 30 cGy, *p* > 0.05; Figure 3C2). As with each prior test, no differences in total object interaction time were observed between the groups, suggesting that impairments in recency memory were not due to decreased interaction, motivation, or locomotor activity. Furthermore, the DI shows that the preference for novelty between the unirradiated male and female groups was comparable (novelty preference, *p* = 0.99; DI, *p* = 0.99; see the Supplementary Materials and Supplementary Table S1). Collectively, data derived from all open arena testing provide convincing evidence for marked sex-dependent differences in behavioral performance following ^4^He irradiation.

### Microglial Activation in Irradiated in Mice

Neuroinflammation has been found to be associated with space radiation-induced cognitive deficits and mood disorders (Sweet et al., 2014; Parihar et al., 2016; Raber et al., 2016). To ascertain whether differences in cognitive deficits found between male and female mice might be due to variations in microglial activity, the number and percentage of activated microglia (CD68+ cells) were quantified in the hippocampus of male (Figures 4A,B) and female (Figures 4D,E) mice 15 weeks following ^4^He ion irradiation. A two-way ANOVA (radiation × sex) analysis on the number of activated microglia demonstrated a significant radiation × sex interaction (*F*_(2,16)_ = 17.66, *p* = 0.001), as well as significant main effect of irradiation (*F*_(1,16)_ = 6.45, *p* = 0.022), but no sex effect (*F*_(1,16)_ = 0.61, *p* = 0.44). *Post hoc* analysis revealed a 2.5-fold increase in the number of activated microglia in male mice when compared to unirradiated control (*p* = 0.001, Figure 4C). Interestingly, the number of activated microglia in female mice was unaffected by irradiation (*p* = 0.99, Figure 4C). Moreover, basal levels of activated microglia were found to be comparable between sexes (*p* = 0.17; Supplementary Materials). In addition, a two-way ANOVA run on the percentage of activated microglia revealed a significant interaction between irradiation and sex (*F*_(2,16)_ = 7.10, *p* = 0.02), as well as significant main effect of sex (*F*_(1,16)_ = 7.10, *p* = 0.02) and irradiation (*F*_(1,16)_ = 4.70, *p* = 0.04). *Post hoc* analysis showed a significant increase in the percentage of activated microglia in the hippocampus of irradiated male mice when compared to controls of the same sex (unirradiated male, *p* = 0.02, Figure 4F), an effect not found in female mice. Overall findings confirm that irradiation had a differential impact on hippocampal microglial activation between the sexes, where male mice showed increased microglial activation compared to females.

### HMGB1 Expression Following ^4^He Ion Irradiation

To identify other inflammatory factors with the potential to influence neurocognitive outcomes, changes in HMGB1 protein were investigated following ^4^He irradiation. Immunohistochemical staining was performed to quantify HMGB1 levels in the hippocampus of both male (Figures 5A,B) and female (Figures 5D,E) mice at 15 weeks following irradiation. Different levels of HMGB1 expression were detected between the control and irradiated mice in both cohorts. Two-way ANOVA demonstrated no significant radiation × sex interaction (*F*_(2,16)_ = 0.99, *p* = 0.33) and no effect of sex (*F*_(1,16)_ = 0.006, *p* = 0.93) but revealed a significant radiation effect (*F*_(1,16)_ = 9.26, *p* = 0.008). As above, the absence of a significant overall sex effect between the groups precluded further *post hoc* analysis; *t*-tests showed a 1.5-fold increase in the number of cells expressing HMGB1 in male mice following irradiation (*p* = 0.01) but did not find any increases in HMGB1 expression in female mice (Figure 5C). Despite the fact that the radiation enhanced the number of HMGB1^+^ cells in male mice, this was not found in female mice (*p* = 0.21). Moreover, we have also determined that irradiation of male mice significantly enhanced the number of microglia that co-expressed HMGB1 (Figure 5F). Two-way ANOVA on the number of microglia co-expressing HMGB1 revealed a significant radiation effect (*p* = 0.01), but no effect of sex (*p* = 0.32), and no significant interaction between sex and irradiation (*p* = 0.18). Independent unpaired *t*-tests performed on female and male mice revealed irradiation to significantly enhance the number of microglia with HMGB1 expression in male mice (*p* = 0.04) but not female mice (*p* = 0.36, Figure 5F).

### TLR4 Expression Following ^4^He Ion Irradiation

To analyze further the consequences of irradiation on inflammatory pathways in the hippocampus, immunohistochemistry was undertaken to measure the levels of TLR4 proteins. TLR4 plays a crucial role in early innate immune responses to inflammatory agents and pathogens and has been implicated in learning and memory processes, stress-induced adaptations, and pathogenesis of neurodegenerative disorders (Paudel et al., 2018). Representative images of TLR4 immunostaining are shown for male (Figures 6A,B) and female mice (Figures 6D,E). Two-way ANOVA demonstrated no significant interaction between radiation and sex (*F*_(2,16)_ = 1.00, *p* = 0.33) as well no main effect of sex (*F*_(1,16)_ = 0.50, *p* = 0.49) but did show significant radiation effect (*F*_(1,16)_ = 8.56, *p* = 0.01). *Post hoc* analysis was not performed as the radiation × sex interaction did not reach significance. Furthermore, the immunoreactivity of TLR4 in the hippocampus of irradiated male mice was increased significantly when compared to their unirradiated controls (unpaired *t*-test, *p* = 0.01, Figure 6C). In contrast, the level of TLR4 in female mice was unaffected by irradiation (unpaired *t*-test *p* = 0.17, Figure 6F). Thus, radiation only had a significant impact on TLR4 expression in male rather than female mice.

### Effect of ^4^He Ion Irradiation on Dendritic Branch Points, Area, and Length

Based on the capability of irradiation to elicit multifaceted neurocognitive complications, we sought to determine whether exposure to ^4^He ions might elicit alterations to the anatomical structure of neurons using a transgenic mouse model that expressed EGFP in certain subsets of neurons. To investigate the possible link between the structural complexity of neurons and cognitive deficits following space irradiation, we focused on the dentate gyrus, a critical structure of the hippocampus involved in learning and memory. For morphological quantification of hippocampal neurons, we measured dendritic branch points, total dendritic length, and area in male and female mice 6 weeks following irradiation. Representative images show dendrites (green) and spines (red) along GCL neurons in male (Figures 7A,B) and female (Figures 7C,D) mice. Two-way ANOVA analysis on dendritic branch points demonstrated no significant interaction between radiation and sex (*F*_(2,8)_ = 0.34, *p* = 0.57) as well as no main effect of sex (*F*_(1,8)_ = 0.98, *p* = 0.35), but revealed a significant radiation effect (*F*_(1,8)_ = 12.09, *p* = 0.008). Similarly, two-way analysis on dendritic area demonstrated no significant interaction between sex and radiation (*F*_(2,8)_ = 2.04, *p* = 0.19) as well as no main effect of sex (*F*_(1,8)_ = 3.28, *p* = 0.11), but did show a significant radiation effect (*F*_(1,8)_ = 6.9, *p* = 0.030). Further, a two-way ANOVA analysis on dendritic length demonstrated no significant interaction between sex and radiation interaction (*F*_(2,8)_ = 0.49, *p* = 0.50) as well as main effect of sex (*F*_(1,8)_ = 1.46, *p* = 0.26), but did show a significant radiation effect (*F*_(1,8)_ = 11.68, *p* = 0.01). A *post hoc* analysis was not performed as the radiation × sex interaction did not reach significance. Furthermore, unpaired *t-tests* revealed that branch points and total dendritic length were decreased significantly in irradiated female mice when compared to their unirradiated controls (branch point, *p* = 0.009, Figure 7E; dendritic length, *p* = 0.02, Figure 7G), an effect not found in irradiated male mice (branch point, *p* = 0.18, Figure 7E; dendritic length, *p* = 0.17, Figure 7G).

### Effect of ^4^He Ion Irradiation on Dendritic Spines

To further explore the possible impact of ^4^He irradiation on neuronal structure, we evaluated dendritic spine number and volume in the molecular layer (ML) of the dentate gyrus in male and female mice. Representative images show dendritic spines (red) along the dendritic shaft (green) in male (Figures 8A,B) and female (Figures 8D,E) mice. Two-way ANOVA analysis on spine number found no significant interaction between radiation and sex (*F*_(2,8)_ = 1.80, *p* = 0.21) as well as no main effect of sex (*F*_(1,8)_ = 2.0, *p* = 0.67), but did show a significant radiation effect (*F*_(1,8)_ = 13.62, *p* = 0.006). Independent *t*-tests performed on female and male mice revealed that irradiation significantly reduced the number of dendritic spines in male (unpaired *t*-test, *p* = 0.0006, Figure 8C) but not female mice (unpaired *t*-test, *p* = 0.29, Figure 8C). Similarly, a two-way ANOVA analysis on spine volume revealed no significant interaction between irradiation and sex (*F*_(2,8)_ = 3.29, *p* = 0.11) as well no significant effect of sex (*F*_(1,8)_ = 5.05, *p* = 0.05), but did show a significant radiation effect (*F*_(1,8)_ = 25.54, *p* = 0.001); *t*-tests revealed that spine volume was decreased significantly in irradiated male mice when compared to their unirradiated controls (unpaired *t*-test, *p* = 0.001, Figure 8F), an effect not found in irradiated female mice (unpaired *t*-test, *p* = 0.14, Figure 8F). Thus, radiation was found to decrease dendritic spine numbers and volume only in male, but not female mice.

## Discussion

The idea that sex can have a significant impact on a variety of neurocognitive outcomes is not new and is supported by numerous clinical and epidemiological studies (Helmstaedter et al., 1999; Torres et al., 2006; Andreano and Cahill, 2009; Urazán-Torres et al., 2013; McCarthy and Wright, 2017; Osborne et al., 2018; Xu et al., 2018). This tenet is also corroborated by space radiation studies in rodents, where male mice have been shown to have an increased susceptibility toward manifesting radiation-induced behavioral decrements as recently reported (Krukowski et al., 2018b). Present findings corroborate these prior results and now show that the heightened sensitivity of male mice exposed to a similar irradiation paradigm is in part due to radiation-induced increases in inflammatory processes involving the HMGB1–TLR4 signaling axis.

The present studies were designed to provide additional mechanistic insight into the potential differences in behavior between the sexes following ^4^He ion exposure. While irradiated female mice showed intact object recognition memory following ^4^He ion exposure, the deficits in NOR were consistent with previous reports in male rodents that have identified alterations in recognition memory following ^4^He irradiation (Rabin et al., 2014; Parihar et al., 2016; Raber et al., 2018). Similar results were obtained between the sexes on the OiP and TO behavioral tasks, where data demonstrated that ^4^He ion irradiation resulted in persistent deficits in object and place recognition memory in male but not female mice. These results corroborate prior findings and add compelling evidence that cosmic radiation exposure has a more severe impact on hippocampal- and cortical-dependent cognitive impairments in males than in females.

Sex differences in neuroinflammation have been explored extensively in both healthy individuals and among those with certain disorders (Spychala et al., 2017; Villa et al., 2018). Until recently, however, virtually no studies have critically evaluated the potential differences in neuroinflammation between the sexes following cosmic irradiation (Krukowski et al., 2018b). To ascertain whether changes in the inflammatory environment of the irradiated brain might provide some clues to sex-dependent differences in behavior, we quantified the yields of inflammatory cell types and cytokine levels. While the number of activated microglia (CD68+) assessed in the hippocampus of female mice was unaffected by irradiation, their basal levels were higher compared to males, whereas in male mice, the number of activated microglia increased over twofold after irradiation compared to controls.

It is now well recognized that activated microglia exert dual functions, commonly referred to as a classical (M1) or “harmful” phenotype and an alternative (M2) or “beneficial” phenotype. M1 activation leads to the release of proinflammatory cytokines (IL-1, IL-12, and IL-18, TNF, and COX2), while M2 activation leads to the enhanced expression of anti-inflammatory pathways and cytokines. A recent report investigating microglia from adult male and female mice found sex-dependent differential gene expression patterns that corresponded with a neuroprotective M2 microglial state in female animals (Villa et al., 2018). One of the key responses of the brain to cosmic radiation exposure is a persistent activation of microglia. As the key innate immune cells of the brain, activated microglial can release a wide range of inflammatory mediators that can be protective over acute times but become toxic over chronic periods of activation (Poulose et al., 2011; Parihar et al., 2016; Perez et al., 2020). Thus, prolonged microglial activation could be a primary cause for many of the behavioral deficits observed at chronic post-irradiation times in male mice. Increased yields of activated M1 microglia may enhance the release of pro-inflammatory factors that exacerbate neuroinflammatory responses. These data are consistent with previous findings where lower phagocytic activity of microglia coincided with improvements in cognition after cosmic radiation exposure (Krukowski et al., 2018a; Allen et al., 2020).

Higher basal levels of activated microglia in unirradiated female compared to male mice may also provide a possible explanation for the muted inflammatory response of female mice to cosmic radiation exposure. The female brain may be pre-conditioned to proinflammatory factors that attenuate radiation responses compared to their male counterparts. This effect may involve the HMGB1 protein, an important proinflammatory cytokine capable of activating and perpetuating inflammation in the brain, in part through interactions with TLR4 receptors (Maroso et al., 2010; Vezzani et al., 2011; Yao et al., 2013; Fujita et al., 2016; Bianchi et al., 2017; Paudel et al., 2018; Okuma et al., 2019; Webster et al., 2019). The capability of radiation exposure to preferentially activate this classic inflammatory signaling pathway in male mice is provocative, and supported by our data showing that immunoreactivity of HMGB1 and TLR4 in the hippocampus was significantly increased in male mice 15 weeks following irradiation, a response that was not observed in female mice. As a type of pattern recognition receptor expressed mainly on microglia, TLR4 responds to exogenous (lipopolysaccharide) and various stress-induced endogenous (HMGB1, heat-shock protein 70) ligands (Yao et al., 2013; Trotta et al., 2014; Brubaker et al., 2015). Past work has demonstrated that TLR4-induced activation of microglia triggers the release of pro-inflammatory molecules responsible for neurotoxic processes in various CNS disorders (Fiebich et al., 2018). Thus, radiation-induced activation of microglia operating through TLR4-dependent mechanisms may injure the neurons by releasing excessive cytokines or stimulating reactive astrocytes (Liddelow et al., 2017) leading to the observed learning and memory deficits in male mice. Further work is nonetheless required to validate the functional and mechanistic role of the HMGB1–TLR4 signaling axis in regulating sex-specific differences in neurocognitive outcomes observed in irradiated male vs. female mice.

Clearly, many factors contribute to radiation-induced brain injury, and increased inflammation and microglial activation likely exacerbate deterioration of neuronal morphology to impair neurotransmission and accelerate cognitive decline. As alluded to above, higher baseline levels of inflammatory cell types may prime the female brain to be more radioresistant, while males exhibit heightened vulnerability to radiation-induced inflammation and normal tissue injury. Different strategies and mechanisms of memory formation may also play a role in mediating the differential susceptibility of male cognitive processes to irradiation (Greene-Schloesser et al., 2012; Balentova and Adamkov, 2015). Although not investigated here, estrogen is likely to impact sex-dependent differences in the irradiated brain. High levels of estrogen in females may provide protection against inflammation and disruptions in neurotransmission that adversely impact cognition. It has been well established that estrogen and its receptors play multiple key neuroprotective roles in preventing a variety of neurodegenerative disorders, including cognitive deficits and mood disorders (Behl and Manthey, 2000; Norbury et al., 2003; Pratap et al., 2016; Song et al., 2018). Increased estrogen has antioxidant and anti-inflammatory properties (Lu et al., 2019) and may also promote axonal and dendritic growth, thereby protecting against the radiation-induced reductions in dendritic morphology and spine density known to occur in male mice subjected to various types of cosmic radiation exposure (Parihar et al., 2015a, 2016).

While space irradiation effects on dendritic morphology variables are well documented in males, the data on females are sparse. For example, low-dose ^56^Fe particles (0.5 Gy, 600 MeV/n) altered hippocampal spine density and dendritic morphology in a region-specific manner, suggesting differential vulnerability in CA1, CA3, and DG regions (Allen et al., 2015) while low-dose ^16^O and ^48^Ti particles reduced dendritic complexity and spine density in the mPFC (Parihar et al., 2015a, 2016). Radiation-induced alterations in dendritic structure and spine density have recently been shown to exhibit sexual dimorphisms (Hinkle et al., 2019). The variability in the female control group for both number and volume of spines is much larger than that seen in the male control group. While reasons for this are uncertain, it may reflect changes in circulating hormone levels, although this remains to be evaluated. Microglia have been found to mediate synaptic loss following irradiation, where CR3-dependent signaling was identified as an underlying mechanism for the enhanced vulnerability of dendritic spines in male mice (Hinkle et al., 2019). While further mechanistic insight is still required to pinpoint the nature of sex-specific changes that differentially impact radiation-induced behavioral changes, such information provides a platform for developing sex-specific treatment strategies for neurological disorders.

## Conclusions

Here, we present further evidence that male and female mice exhibit divergent behavioral responses following low dose exposure to ^4^He ions. Adverse neurocognitive outcomes were tracked with increased radiation-induced microglial activation and HMGB1 and TLR4 expression, responses that were not found in the irradiated female rodent brain. Future studies will now be focused on elucidating more precisely the nature of these sex-dependent vulnerabilities to cosmic radiation exposures, with an emphasis on the interactions between microglia and other neural cell types. This research will be critical in evaluating potential countermeasures, which will likely require careful consideration of how sex-specific responses affect the brain and behavior following deep space radiation exposure.

## Data Availability Statement

All datasets generated for this study are included in the article/Supplementary Material.

## Ethics Statement

The animal study was reviewed and approved by the Institutional Animal Care and Use Committee at the University of California, Irvine and NASA Space Radiation Laboratory (NSRL) at Brookhaven National Laboratory.

## Author Contributions

VP and CL conceptualized the idea, contributed to study design, result interpretation, analysis, manuscript writing, and proofreading. MA, BA, and AS performed all the behavioral studies. MU, XL, AA, LF, and EP collected immunohistochemistry and structural data. EG helped in collecting structural analysis and confocal microscopy. All authors contributed to the article and approved the submitted version.

## Conflict of Interest

The authors declare that the research was conducted in the absence of any commercial or financial relationships that could be construed as a potential conflict of interest.
