# Supplementary material for: Sex-Specific Cognitive Deficits Following Space Radiation Exposure
Source: Front Behav Neurosci. 2020 Sep 16;14:535885. doi: 10.3389/fnbeh.2020.535885 (PMC7525092; doi:10.3389/fnbeh.2020.535885)
Supplement: Supplementary file 1 [file Data_Sheet_1.pdf]

Sex Specific Cognitive Deficits Following Space Radiation Exposure

Vipan K. Parihar*, Maria C. Angulo, Barrett D. Allen, Amber Syage, Manal T. Usmani, Estrella Passerat de la Chapelle, Amal Nayan Amin, Lidia Flores, Xiaomeng Lin, Erich Giedzinski, Charles L. Limoli*

Department of Radiation Oncology, University of California, Irvine, CA 92697-2695, USA

***Corresponding authors:**

Dr. Vipan K. Parihar
Department of Radiation Oncology
University of California, Irvine
Irvine CA 92697-2695, USA. Phone: (949) 824-7395
E-mail: vipank@uci.edu

Prof. Charles L. Limoli
Department of Radiation Oncology
University of California, Irvine
Irvine CA 92697-2695, USA. Phone: (949) 824-3053
E-mail: climoli@uci.edu

Supplemental Material

Animals: All animal procedures were carried out in accordance with National Institutes of Health and Institutional Animal Care guidelines and were approved by the Institutional Animal Care and Use Committee at the University of California, Irvine (UCI) and NASA Space Radiation Laboratory (NSRL) at Brookhaven National Laboratory. Six-month-old male and female C57BL/6J mice acquired from the Jackson Laboratory (stock # 000664) were used for the behavior and immunohistochemical follow up studies beginning 12 weeks following irradiation. To evaluate the structural changes in the dentate gyrus neurons following irradiation, six-month-old transgenic male and female mice [strain Tg(Thy1-EGFP) MJrsJ, Stock No. 007788, Jackson Laboratory, CT, USA] harboring the Thy1-EGFP transgene were used. Thy1-EGFP transgene mice were bred at UCI vivarium, genotyped and mice positive for the Thy1-EGFP transgene were used to determine any sex-specific changes to dendritic structure of dentate gyrus neurons 6 weeks following irradiation (Parihar et al. 2016; Parihar et al. 2015b; Parihar and Limoli 2013). Animals were maintained in standard housing conditions (20 °C ± 1 °C; 70% ± 10% humidity; 6:00Am - 6:00 Pm light and dark cycle,) and provided ad libitum access to standard rodent chow and water. Mice were group housed (3-4 mice per cage) in Individually Ventilated Caging (IVC) systems (Techniplast IVC) using 1/8" corncob bedding.

NSRL Beam: Charged particles (^{4}He) at 400 MeV/n were generated and delivered at the NASA Space Radiation Laboratory (NSRL) at Brookhaven National Laboratory using a dose rate of 5cGy/min. NSRL physicists have optimized the parameters required to deliver a uniform beam purity (≥ 95%) and distribution to the target area(La Tessa et al. 2016)

Behavioral testing: Novel Object Recognition (**NOR**), Object-in-Place (**OiP**) tests data were collected from two cohorts (cohort 1, n =8 per group; cohort 2, n=4 per group), while Temporal Order (**TO)** tests were collected from a single cohort (cohort 1, n =8 per group). All the behavior tests were performed 12 weeks post irradiation during the light period of the day in the rooms with illumination 915 Lux. Data collections and analysis were done by two independent observers who were blinded to the identity of animal groups.

NOR, OiP and TO tasks interrogate functional connectivity between different regions of the brain including the prefrontal cortex (mPFC), perirhinal cortex (PRC) and hippocampus. NOR is a measure of preference for novelty, which relies on intact mPFC function, while OiP is a test of associative recognition memory that depends on interactions between the hippocampus, mPFC and PRC functions (Barker et al. 2007; Barker and Warburton 2015). The TO task quantifies recency discrimination to previously explored objects (Barker et al. 2007; Barker and Warburton 2015). Behavior tests were conducted as described previously (Acharya et al. 2017; Acharya et al. 2016; Barker et al. 2007; Barker and Warburton 2015; Parihar et al. 2016; Parihar et al. 2015a; Parihar et al. 2015b). All the behavior tests were conducted in a dimly lit (rooms illumination 915 Lux.), dedicated room separated from housing. Four arena boxes made of acrylic (30x30x30 cm) were placed two by two, layered with fresh, autoclaved, corncob bedding, and placed on the floor. Care was taken to keep the arenas in exactly the same location, and mice were never rotated to other arenas during the duration of the behavior testing. A camera was mounted above the arenas to record each trial. Each arena was thoroughly cleaned between trials with 70% ethanol, and fresh bedding was used to eliminate distinguishing olfactory cues (Acharya et al. 2017; Acharya et al. 2019; Parihar et al. 2016; Parihar et al. 2015a; Parihar et al. 2015b; Parihar et al. 2018; Parihar et al. 2015c).

NOR test: The NOR test was administered after 3 days of habituation (10 min/day). Objects to be distinguished were made of a similar plastic material but varied in color, shape, and size. To ensure that objects were in exactly the same position for each trial, magnets were used to hold them in place ≈ 7 cm from opposing corners and 16 cm apart. During the testing day, mice were exposed to two duplicate objects within the arena for 5 min (familiarization). They were then allowed to rest for 5 min in their home cage within the behavioral room while the objects were cleaned (70% ethanol), and a novel object was added. Mice were then returned to the arena for 5 min, where they explored the familiar and novel object (testing). The objects were counterbalanced between the groups and these objects were not used in subsequent testing. Trials were later hand scored by an individual blinded to the experimental groups and calculated by using the discrimination index. A positive score is counted when the nose of the mouse is within 1 cm and pointed in the direction of the object. Time was not scored for mice that were near but not facing the object. The novel object discrimination index was also directly compared between controls and irradiated mice. Additionally, the total time exploring both or all the objects in test phase was examined and compared between the groups to ascertain whether depression (or lack of motivation) or reduced locomotor activity interfered with the place or object recognition memory testing.

OiP task: The week after cessation of NOR testing, mice were habituated again to their arenas (10min/day) for 2 consecutive days. On the third day, mice were exposed to four objects of varying size, color, and shape for 5 min (familiarization). Mice were then allowed to rest for 5min in their home cage within the same room as the arenas. Objects were cleaned with 70% ethanol, and two of the four objects switched locations. The objects were counter balanced between the groups to assure there were no inherent preferences to a particular object. Mice were returned to the arenas for 5min of exploration (testing). Trials were later hand scored by an individual blinded to the experimental groups and calculated by using the discrimination index as described earlier. Positive scores were calculated based on the same criteria detailed for the NOR task earlier.

TO task: For the TO task, animals were habituated (10 min) 1 d before testing. On the day of testing, animals were placed in the arena with two identical objects for 5 min (familiarization). Animals were reintroduced (4 h later) in the same arena and allowed to familiarize (5 min) with two new (but identical) objects placed in the exact same positions. Animals were finally reintroduced (1 h later) for temporal order memory testing (5 min) of the prior arrangement of objects, in which one was substituted for the original object, presented 5 h before. Trials were later hand scored by an individual blinded to the experimental groups and calculated by using the discrimination index as described earlier. Positive scores were calculated based on the same criteria detailed for the earlier tasks.

Tissue processing and immunohistochemistry:

For immunohistochemical analysis, 15 weeks following ^{4}He irradiation, mice were euthanized and perfused with 4% paraformaldehyde (Acros Organics), and brain tissues were processed for coronal sectioning using a cryostat (Leica Microsystems). Briefly, each animal was first deeply anesthetized with isoflurane in a Plexiglas chamber until it ceased respiration. Following this, the chest was surgically opened, the heart was exposed, and a nick was made in the right atrium, and sequentially infused with normal saline and 4% paraformaldehyde through the left ventricle. The brains were dissected, post-fixed overnight in 4% paraformaldehyde solution, washed in PB, and treated with different concentrations (10–30%) of sucrose gradually until they sank to the bottom

of the container. Each brain was then mounted on a cryostat chuck and 30-micrometer thick coronal sections through the hippocampus were cut and collected serially in 24-well plates. Three sets of serial sections (every 10th) through the entire hippocampus were picked from each animal belonging to control and irradiated groups and processed for CD68 or HMBG1 or TLR4 immunostaining. Sections were washed in PBS (pH 7.4), blocked for 30 min in 4% (wt/vol) BSA and 0.1% TritonX 100 (TTX). Afterwards, primary antibodies including rabbit rat anti-mouse CD68 (1:500, AbD Serotec; MAC 1957GA), rabbit anti-HMGB1 (1:500, Abcam, AB 18256) or mouse anti-TLR4(1:200, Novus, NB100-56566) were used for incubation overnight at 4 °C containing 1% BSA, 0.1% TTX. Secondary antibodies such as Alexa Fluor 594 goat anti-rat IgG (1:500, Invitrogen), Alexa Fluor 650 goat anti-rabbit IgG (1:500, Invitrogen,) Alexa Fluor 650 goat anti-mouse IgG (Invitrogen USA) were applied for 1 h at room temperature, and then counterstained using DAPI and mounted for histochemical analysis. Immunofluorescent sections were imaged using Nikon Eclipse Ti C2 microscope to obtain 20 to 30 z-stacks (1024 × 1024 pixels, 0.5 µm each) using 40× PlanApo oil-immersion lens (Nikon). For quantification of CD68+ cells and HMGB1+ cells, 3D deconvolution and reconstruction was carried out using the AutoQuantX3 algorithm (MediaCybernetics). Deconvolution combined with 3D reconstruction yields higher spatial resolution images for the immunofluorescent cell bodies. Analysis of TLR4 was performed using the IMARIS volume tool (v7.6, Bitplane Inc., Switzerland) that detects immunostained puncta within 3D deconvoluted image stacks. Puncta satisfying pre-defined criteria (verified visually for accuracy) were converted to surface for quantification under preset parameters kept constant throughout subsequent analyses. (Acharya et al. 2019; Acharya et al. 2016; Parihar et al. 2016; Parihar et al. 2015a; Parihar et al. 2015b).

Confocal microscopy, imaging and image processing for structural analysis: The expression of EGFP reveals the structure of the neuron, including the cell body, axonal and dendritic arbors. It is also sufficiently bright to reveal detailed structures such as axonal boutons and dendritic spines. In previous quantitative studies we demonstrated that radiation reduce the dendritic complexity of hippocampal granular cell layers following proton or gamma irradiation (Parihar and Limoli 2013; Parihar et al. 2015c), and low dose ^{16}O and ^{48}Ti particles reduced dendritic complexity and spine density in the medial prefrontal cortex(Parihar et al. 2016; Parihar et al. 2015b). In this study we decided to extend this effect to the dentate gyrus subarea of the hippocampus using the same morphometric procedures to determine if irradiation alters dendritic morphology in a sex-dependent manner. For dendritic analyses, 100 µm-thick hippocampus sections were prepared for confocal imaging. In each cohort three mice per group were used, and 3 tissues per animal (total 9 tissues /group) were used to generate Z-stacks using a Nikon Eclipse Ti C2 microscope. Images comprising each Z-stack (1024 × 1024 pixels) were acquired at (60x) over the entire dendrite tree at 0.1 µm increments. To cover the neuron with entire morphometric ending and braches, the neurons were imaged two different overlapping Z-stacks. These images (Z-stacks) were then stitched together and Z-compressed using XuvStitch 8.1X64 (XuvTools). Quantification of dendritic parameters was derived from Z-stacks reconstructed in 3D from deconvoluted images using the AutoQuantX3 algorithm (MediaCybernetics, MD, USA). Deconvoluted 3D reconstructions yielded high spatial resolution images for detailed dendritic tracing and spine classification using the IMARIS software suite (Bitplane Inc.) as previously described (Montay-Gruel et al. 2019; Parihar et al. 2016; Parihar et al. 2015b).

Neuron reconstruction and spine classification: Details regarding the reconstruction of neurons and spines quantification have been described (Montay-Gruel et al. 2019; Parihar et al. 2016; Parihar et al. 2015b). Briefly, an algorithm for tracing dendritic filaments was used to

reconstruct the entire dendritic tree spanning a series of Z-stacks (210×210μm^2). Dendritic tracing originates from the soma (diameters 90-100μm) and terminates once dendrite diameters reach sizes 0.6 μm. Reconstructed dendritic trees are then reanalyzed for dendritic spines that can be labeled, manually verified, morphologically categorized and quantified. For spines to be included in our analyses a maximum spine length and minimum spine end diameter were set at 3 and 0.3 μm, respectively. Parameters for the reconstruction and classification of dendrites and spines were selected after manual measurements from multiple neurons (10–15) from each cohort. Parameters were further validated from an independent series of pilot reconstructions in both manual and semi-automatic modes. Images were compared for accuracy and consistency to ensure that selected parameters represented actual variations in dendritic structure.

Table 1: Discrimination indices calculated for sham irradiated male and female mice were comparable, indicating a similar preference for novelty.

**End point Assessment**	**Outcome measure**	**Sham irradiated**		**Statistics**
		**Male**	**Female**	
NOR (Fig.1)	Discrimination index	26.52 ± 2.82	21.02 ± 4.45	p > 0.05
	Time spent with novel object (%)	63.26 ± 1.41	60.5 ± 2.23	P > 0.05
OIP (Fig. 2)	Discrimination index	21.00 ± 6.07	21.13 ± 3.11	p > 0.05
	Time spent with novel object (%)	60.45 ± 3.04	60.55 ± 1.55	P > 0.05
TO (Fig. 3)	Discrimination index	38.06 ± 3.38	28.13 ± 4.58	P > 0.05
	Time spent with novel object (%)	69.03 ± 1.69	64.16 ± 2.29	P > 0.05
Activated Microglia (Fig. 4)	CD68+ cells	21.40 ± 2.81	35.80 ± 2.73	P > 0.05

References:

Acharya, M. M., A. A. Baddour, T. Kawashita, B. D. Allen, A. R. Syage, T. H. Nguyen, N. Yoon, E. Giedzinski, L. Yu, V. K. Parihar, and J. E. Baulch. 2017. "Epigenetic determinants of space radiation-induced cognitive dysfunction." *Sci Rep* 7:42885.

Acharya, M. M., J. E. Baulch, P. M. Klein, A. A. D. Baddour, L. A. Apodaca, E. A. Kramar, L. Alikhani, C. Garcia, Jr., M. C. Angulo, R. S. Batra, C. M. Fallgren, T. B. Borak, C. E. L. Stark, M. A. Wood, R. A. Britten, I. Soltesz, and C. L. Limoli. 2019. "New Concerns for Neurocognitive Function during Deep Space Exposures to Chronic, Low Dose-Rate, Neutron Radiation." *eNeuro* 6(4).

Acharya, M. M., K. N. Green, B. D. Allen, A. R. Najafi, A. Syage, H. Minasyan, M. T. Le, T. Kawashita, E. Giedzinski, V. K. Parihar, B. L. West, J. E. Baulch, and C. L. Limoli. 2016. "Elimination of microglia improves cognitive function following cranial irradiation." *Sci Rep* 6:31545.

Barker, G. R., F. Bird, V. Alexander, and E. C. Warburton. 2007. "Recognition memory for objects, place, and temporal order: a disconnection analysis of the role of the medial prefrontal cortex and perirhinal cortex." *J Neurosci* 27(11):2948-57.

Barker, G. R., and E. C. Warburton. 2015. "Object-in-place associative recognition memory depends on glutamate receptor neurotransmission within two defined hippocampal-cortical circuits: a critical role for AMPA and NMDA receptors in the hippocampus, perirhinal, and prefrontal cortices." *Cereb Cortex* 25(2):472-81.

La Tessa, C., M. Sivertz, I. H. Chiang, D. Lowenstein, and A. Rusek. 2016. "Overview of the NASA space radiation laboratory." *Life Sci Space Res (Amst)* 11:18-23.

Montay-Gruel, P., M. M. Acharya, K. Petersson, L. Alikhani, C. Yakkala, B. D. Allen, J. Ollivier, B. Petit, P. G. Jorge, A. R. Syage, T. A. Nguyen, A. A. D. Baddour, C. Lu, P. Singh, R. Moeckli, F. Bochud, J. F. Germond, P. Froidevaux, C. Bailat, J. Bourhis, M. C. Vozenin, and C. L. Limoli. 2019. "Long-term neurocognitive benefits of FLASH radiotherapy driven by reduced reactive oxygen species." *Proc Natl Acad Sci U S A* 116(22):10943-51.

Parihar, V. K., B. D. Allen, C. Caressi, S. Kwok, E. Chu, K. K. Tran, N. N. Chmielewski, E. Giedzinski, M. M. Acharya, R. A. Britten, J. E. Baulch, and C. L. Limoli. 2016. "Cosmic radiation exposure and persistent cognitive dysfunction." *Sci Rep* 6:34774.

Parihar, V. K., B. D. Allen, K. K. Tran, N. N. Chmielewski, B. M. Craver, V. Martirosian, J. M. Morganti, S. Rosi, R. Vlkolinsky, M. M. Acharya, G. A. Nelson, A. R. Allen, and C. L. Limoli. 2015a. "Targeted overexpression of mitochondrial catalase prevents radiation-induced cognitive dysfunction." *Antioxid Redox Signal* 22(1):78-91.

Parihar, V. K., B. Allen, K. K. Tran, T. G. Macaraeg, E. M. Chu, S. F. Kwok, N. N. Chmielewski, B. M. Craver, J. E. Baulch, M. M. Acharya, F. A. Cucinotta, and C. L. Limoli. 2015b. "What happens to your brain on the way to Mars." *Sci Adv* 1(4).

Parihar, V. K., and C. L. Limoli. 2013. "Cranial irradiation compromises neuronal architecture in the hippocampus." *Proc Natl Acad Sci U S A* 110(31):12822-7.

Parihar, V. K., M. Maroso, A. Syage, B. D. Allen, M. C. Angulo, I. Soltesz, and C. L. Limoli. 2018. "Persistent nature of alterations in cognition and neuronal circuit excitability after exposure to simulated cosmic radiation in mice." *Exp Neurol* 305:44-55.

Parihar, V. K., J. Pasha, K. K. Tran, B. M. Craver, M. M. Acharya, and C. L. Limoli. 2015c. "Persistent changes in neuronal structure and synaptic plasticity caused by proton irradiation." *Brain Struct Funct* 220(2):1161-71.
